# Altered Gray Matter Volume and Resting-State Connectivity in Individuals With Internet Gaming Disorder: A Voxel-Based Morphometry and Resting-State Functional Magnetic Resonance Imaging Study

**DOI:** 10.3389/fpsyt.2018.00077

**Published:** 2018-03-27

**Authors:** Ji-Woo Seok, Jin-Hun Sohn

**Affiliations:** ^1^Department of Counseling Psychology, Honam University, Gwangju, South Korea; ^2^Department of Psychology, Brain Research Institute, Chungnam National University, Daejeon, South Korea

**Keywords:** Internet gaming disorder, voxel-based morphometry, resting-state functional magnetic resonance imaging, functional connectivity, middle frontal gyrus, caudate nucleus

## Abstract

Neuroimaging studies on the characteristics of individuals with Internet gaming disorder (IGD) have been accumulating due to growing concerns regarding the psychological and social problems associated with Internet use. However, relatively little is known about the brain characteristics underlying IGD, such as the associated functional connectivity and structure. The aim of this study was to investigate alterations in gray matter (GM) volume and functional connectivity during resting state in individuals with IGD using voxel-based morphometry and a resting-state connectivity analysis. The participants included 20 individuals with IGD and 20 age- and sex-matched healthy controls. Resting-state functional and structural images were acquired for all participants using 3 T magnetic resonance imaging. We also measured the severity of IGD and impulsivity using psychological scales. The results show that IGD severity was positively correlated with GM volume in the left caudate (*p* < 0.05, corrected for multiple comparisons), and negatively associated with functional connectivity between the left caudate and the right middle frontal gyrus (*p* < 0.05, corrected for multiple comparisons). This study demonstrates that IGD is associated with neuroanatomical changes in the right middle frontal cortex and the left caudate. These are important brain regions for reward and cognitive control processes, and structural and functional abnormalities in these regions have been reported for other addictions, such as substance abuse and pathological gambling. The findings suggest that structural deficits and resting-state functional impairments in the frontostriatal network may be associated with IGD and provide new insights into the underlying neural mechanisms of IGD.

## Introduction

Online gaming provides enjoyment and relieves stress, in addition to many other advantages. Consequently, the number of Internet gamers has consistently increased worldwide. Excessive Internet gaming can, however, limit real-life experience, resulting in various negative psychosocial consequences (1–3). Internet Gaming Disorder (IGD) is defined as a compulsive and pathological use of devices enabling access to the Internet and has serious negative consequences. Section III of the Diagnostic and Statistical Manual of Mental Disorders-5 (DSM-5) states that IGD is a condition that requires more clinical research (4).

Recently, neuroimaging studies on IGD have investigated functional and structural alterations in the brain to identify the neuronal correlates related to the development of IGD (5). Task-related functional magnetic resonance imaging (fMRI) has revealed functional disturbances in individuals with IGD (2, 6, 7–11). The results of these fMRI studies indicate that during exposure to computer games, video games, or online games, individuals with IGD, as compared with healthy controls (HC), show an increased craving for gaming as well as altered brain activity in various regions such as the caudate nucleus, dorsolateral prefrontal area, nucleus accumbens, anterior cingulate cortex, and hippocampus (7–10).

Although task-based fMRI studies can identify specific functional disturbances within individuals with IGD, evaluation of resting-state functional connectivities may provide different and potentially broader significance (12). Resting-state fMRI is a method for evaluating functional connections and interactions between regions during a task-free condition. Assessment of the resting-state fMRI network can provide more information about distributed circuit abnormalities in neuropsychiatric illnesses (13, 14). Resting-state fMRI studies of IGD have been conducted to identify the specific neurobiological network underlying reward and cognitive processes in terms of functional connectivity (15–18). These studies have reported enhanced functional connectivity or regional homogeneity in the middle temporal gyrus and the cerebellum (15, 16, 18). Moreover, Hong et al. (17) observed decreased functional connectivity in subcortical brain regions.

Mounting evidence from structural brain imaging studies have revealed that IGD might be linked to possible structural changes within the brain (17, 19–22). The most widely used morphometric analysis methods for brain analysis are volume-based gray matter (GM) measurements such as voxel-based morphometry (VBM) and surface-based cortical thickness measurements using FreeSurfer (23). Han et al. (19) and Weng et al. (21) investigated structural abnormalities in the brain of adolescents with IGD using VBM and reported reduced GM volumes in the orbitofrontal cortex, insula, temporal gyrus, and occipital cortex. Studies evaluating cortical thickness to observe structural changes in the brains of individuals with IGD have revealed decreased cortical thickness in the orbitofrontal cortex, insula, parietal cortex, and postcentral gyrus (17, 22).

More recently, a combined structural and functional MRI study reported a negative correlation between impulsivity and left amygdala volume, and lower functional connectivity between the amygdala and the dorsolateral prefrontal cortex (DLPFC) (13, 14). These results suggest that altered GM volume and functional connectivity in the amygdala might be related to impulsivity and represent a vulnerability to IGD (13, 14). Two studies recently assessed the compatibility difference in both brain structure and functional connectivity. First, Jin et al. (24) found that individuals with IGD had significantly decreased GM volume in the prefrontal cortex, including the DLPFC, orbitofrontal cortex, anterior cingulate cortex, and supplementary motor area, and decreased functional connectivity in the prefrontal striatal circuit. Second, Yuan et al. (25) found decreased striatum volume and resting-state functional connectivity differences in the frontostriatal circuits between individuals with IGD and HC. These results suggest that at the circuit level, IGD may share similar neural mechanisms with substance use disorder (24, 25).

In conclusion, the results of previous studies and recent reviews using neuroimaging techniques suggest that IGD is related to neuroanatomical alterations in frontostriatal circuits, similar to substance use disorder (7–11, 24–28). Moreover, the similarity of psychopathological symptoms and neural processes between IGD and substance use disorder suggests a possible shared vulnerability mechanism (9, 27, 28).

To date, few studies have been conducted on functional and structural alterations in IGD using structural combined with resting-state functional network analyses (13, 14, 25, 29). Moreover, these studies of IGD did not eliminate the influence of behavior characteristics (i.e., average gaming hours) on the relationship between IGD and brain alteration although repeated behaviors could change the brain structure (30). Therefore, to strengthen the attribution of IGD characteristics including psychiatric disorder (i.e., addiction) to the brain alteration, we controlled for the effect of gaming activity on the changes of brain structure and connectivity in IGD.

In this study, we examined alterations in structure and functional connectivity in the brains of individuals with IGD, using 3 T magnetic resonance imaging of the brain GM volume and resting-state connectivity analysis. Specifically, we investigated whether the GM volume is altered in the frontostriatal circuits of individuals with IGD, and whether a reduction in GM volume is associated with altered functional connectivity. We also identified whether these alterations were exhibited after excluding gaming activity.

## Materials and Methods

### Participants and Measurement Instruments

Twenty right-handed male participants with IGD (age range: 20–26 years) were recruited *via* broadcasting online bulletin boards and among individuals attending an Internet addiction treatment center, a cyber addiction information center, or local Internet addiction recovery group meetings. All participants in the IGD group were interviewed by two qualified psychiatrists, according to the diagnostic criteria for IGD outlined in the Diagnostic and Statistical Manual of Mental Disorders-5 (31). Using the same criteria, 20 age- and sex-matched HC (age range: 20–27 years) were also recruited. None of the participants fulfilled the criteria for any other psychiatric or neurological disorder such as schizophrenia, anxiety, depression, gambling addiction, or substance dependence. None of the participants reported any previous experience with gambling or illicit drugs.

All participants provided their written informed consent after being thoroughly informed about the details of the experiment. The Chungnam National University Institutional Review Board approved the experimental and consent procedures (approval number: P01-201602-11-002). All participants received financial compensation (50 US dollars) for their participation.

Participants completed a survey containing questions regarding their demographic characteristics and Internet gaming activities within the past 12 months, such as “In the past year, on average, about how many days per week did you play Internet games?” and “In the past year, on average, about how many minutes per day did you spend on an Internet game?” In addition, standardized scales such as the Barratt Impulsiveness Scale-II [BIS (32)], Alcohol Use Disorders Identification Test (33), and the Beck Depression Inventory [BDI (34)] were used to assess the psychological characteristics of the participants.

The severity of IGD was measured using Young’s online Internet addiction test (IAT) (35). The IAT is a reliable and valid instrument for classifying Internet addiction disorder (36). The IAT comprises a total of 20 questions that are designed to assess compulsive Internet use, withdrawal symptoms, psychological dependence, and related problems in daily life. Ratings were made based on a 5-point scale, ranging from 1 (never) to 5 (very). The score ranges from 20 to 100, and a total score of 50 or higher indicates occasional or frequent Internet-related problems due to uncontrolled Internet usage (http://netaddiction.com/internet-addiction-test/).

### Data Acquisition

A 3.0 T MRI scanner (Achieva Intera 3 T; Philips Healthcare, Best, the Netherlands) was used for image acquisition. T1-weighted anatomical images were acquired using the following parameters: repetition time = 280; echo time = 14 ms; flip angle = 60°; field of view = 24 cm × 24 cm; matrix = 256 × 256; slice thickness = 4 mm. During resting-state scanning, 180 images were acquired with a single-shot, echo-planar pulse sequence (repetition time = 2,000 ms; echo time = 28 ms; slice thickness = 4 mm, no gap; matrix = 64 × 64; field of view = 24 cm × 24 cm; and flip angle = 80°). The participants were instructed to keep their eyes closed comfortably, to stay awake, not to think of anything, and not to sleep or doze off during resting-state scanning. After the scan, all participants were asked whether they had stayed awake with their eyes closed during the entire scanning time. Data from participants who reported difficulties in staying fully awake were discarded and not used for any further analysis.

### VBM Analysis

Voxel-based morphometry analysis was performed using SPM8 software (http://www.fil.ion.ucl.ac.uk/spm) and the VBM8 toolbox (http://dbm.neuro.uni-jena.de/vbm.html). MR images were processed using the diffeomorphic non-linear registration algorithm (diffeomorphic anatomical registration through exponentiated lie algebra, DARTEL) technique to improve intersubject brain image registration (37). Briefly, the VBM analysis consisted of the following four steps: (1) MR images were segmented into GM, white matter (WM), and cerebrospinal fluid; (2) customized GM templates were created from the study images using the DARTEL technique; (3) after a linear affine registration of the GM DARTEL templates to the tissue probability maps in Montreal Neurological Institute (MNI) space, non-linear warping of GM images was applied to the DARTEL GM template and then used in the modulation step to guarantee that the relative amount of GM volumes was preserved following the spatial normalization procedure; (4) modulated GM images were smoothed using an 8-mm full width at half maximum Gaussian kernel for statistical analyses.

After preprocessing, GM volume was compared between individuals with IGD and HC. An absolute threshold mask of 0.1 was used for GM analyses to avoid possible edge effects around the border between the gray and WM.

To control for extraneous effects of age, years of education, impulsivity, and depression, these variables were added as covariates. We also conducted between group analysis by adding the average gaming hours as a covariate to identify the effect of IGD as excluding the influence of behavior characteristics related to IGD.

In each group, partial correlation analyses were performed to investigate the association between GM volume and the severity of IGD (i.e., the score of IAT) by excluding the extraneous variables (i.e., age, years of education, impulsivity, and depression). Furthermore, another partial correlation analysis was performed by controlling the extraneous variables with an additional covariate (i.e., the average gaming hours). The statistical significance of group differences was set at *p* < 0.05, corrected for multiple comparisons using the false discovery rate (FDR) method, at a cluster extent of >50 voxels.

### Functional Connectivity Analysis

Functional connectivity analysis was performed using the CONN functional connectivity toolbox v.15 [http://www.nitrc.org/projects/conn; cited in Whitfield-Gabrieli et al. (38)] to identify resting-state properties in structurally altered brain regions. Resting-state data were first preprocessed using standard preprocessing steps, including slice-time correction, motion correction with artifact rejection, spatial normalization to the standardized brain space using the template image, and smoothing with an 8-mm isotropic Gaussian kernel. Before subject-level analysis, denoising procedures were performed on the data using the BOLD (blood-oxygen-level dependent) signal derived from WM masks and cerebral spinal fluid, and motion correction parameters from the realignment stage of the spatial preprocessing, as covariates of no interest in a linear regression model. Then, a band-pass filter between 0.01 and 0.08 Hz was applied to the time series to extract the specific frequency area signal related to nerve cell activity.

After preprocessing and denoising procedures, the functional connectivity analysis was carried out by applying a seed-based approach by choosing the left caudate nucleus cluster peak from the VBM analysis, (−9 +8 +15) in MNI space. We chose the left caudate nucleus as the seed region of interest for the subsequent functional connectivity analysis because the left caudate nucleus was linked to IGD severity in the VBM analysis, and because previous studies revealed functional and structural alterations in the left caudate nucleus within individuals with IGD (24, 25). The cross-correlation coefficient between these seed voxels and all other voxels was calculated to generate a correlation map. For second-level analyses, correlation coefficients were transformed into normally distributed *z*-scores using a Fisher transformation. Age, years of education, impulsiveness, and depression were added as covariates in the second-level analyses. For group-level comparisons, two-sample *t*-tests were performed to compare *z*-value maps between individuals with IGD and HC, with a height threshold of an uncorrected *p* < 0.001 and an extent threshold of an FDR-corrected *p* < 0.05 at the cluster level. ANCOVA was also conducted with adding the average gaming hours as a covariate to identify the difference between groups as excluding the influence of behavior characteristics related to IGD.

Within each group, partial correlation analyses between the severity of IGD (i.e., IAT) and the average *z*-scores of brain regions exhibiting reduced functional connectivity with the left caudate nucleus were performed to examine the relationship between IGD severity and altered functional connectivity with excluding the extraneous variables (i.e., age, years of education, impulsivity, and depression). Another partial correlation was also performed by adding the average gaming hours as a covariate with the extraneous variables.

### Correlation Analysis Between Brain Structure and Functional Connectivity

To investigate the association between structure and functional connectivity in the left caudate nucleus of individuals with IGD, a correlation analysis was performed after statistically controlling for impulsiveness and depression.

## Results

### Participant Characteristics

As shown in Table 1, individuals with IGD and HC did not differ significantly in age (*t* = 0.83, *p* > 0.05) and education duration (*t* = 0.67, *p* > 0.05). However, relative to HC, individuals with IGD scored higher on measures of average gaming hours per day (*t* = 7.25, *p* < 0.001) and average gaming days per week (*t* = 7.42, *p* < 0.001), and had higher IAT scores (*t* = 11.37, *p* < 0.001). Individuals with IGD were also more depressed (*t* = 4.88, *p* < 0.001) and impulsive (*t* = 5.23, *p* < 0.001) than controls. Internet addiction scores were positively associated with depression scores (*r* = 0.71, *p* < 0.001) and impulsiveness scores (*r* = 0.66, *p* < 0.001).

**Table 1:** Demographic and clinical characteristics of the IGD group and HC.

Variables (mean ± SD)	IGD	HC	*t*
Age (years)	21.70 ± 2.74	22.40 ± 2.62	0.83
Education (years)	14.55 ± 2.93	15.15 ± 2.72	0.67
Average gaming hours per day	11.87 ± 5.33	1.90 ± 3.06	7.25***
Average gaming days per week	6.75 ± 0.71	2.4 ± 2.52	7.42***
AUDIT score	4.73 ± 3.07	3.75 ± 2.59	1.09
BDI score	12.4 ± 7.36	3.3 ± 3.89	4.88***
BIS-II score	56.00 ± 5.34	47.50 ± 4.92	5.23***
IAT score	71.85 ± 12.82	29.80 ± 8.80	12.09***

*BDI, Beck Depression Scale; BIS, Barrett’s Impulsiveness Scale-II; IGD, Internet gaming disorder; IAT, Internet addiction test; HC, healthy controls*.

***p < 0.001 for group comparisons.

### VBM Analysis

**Figure 1:**
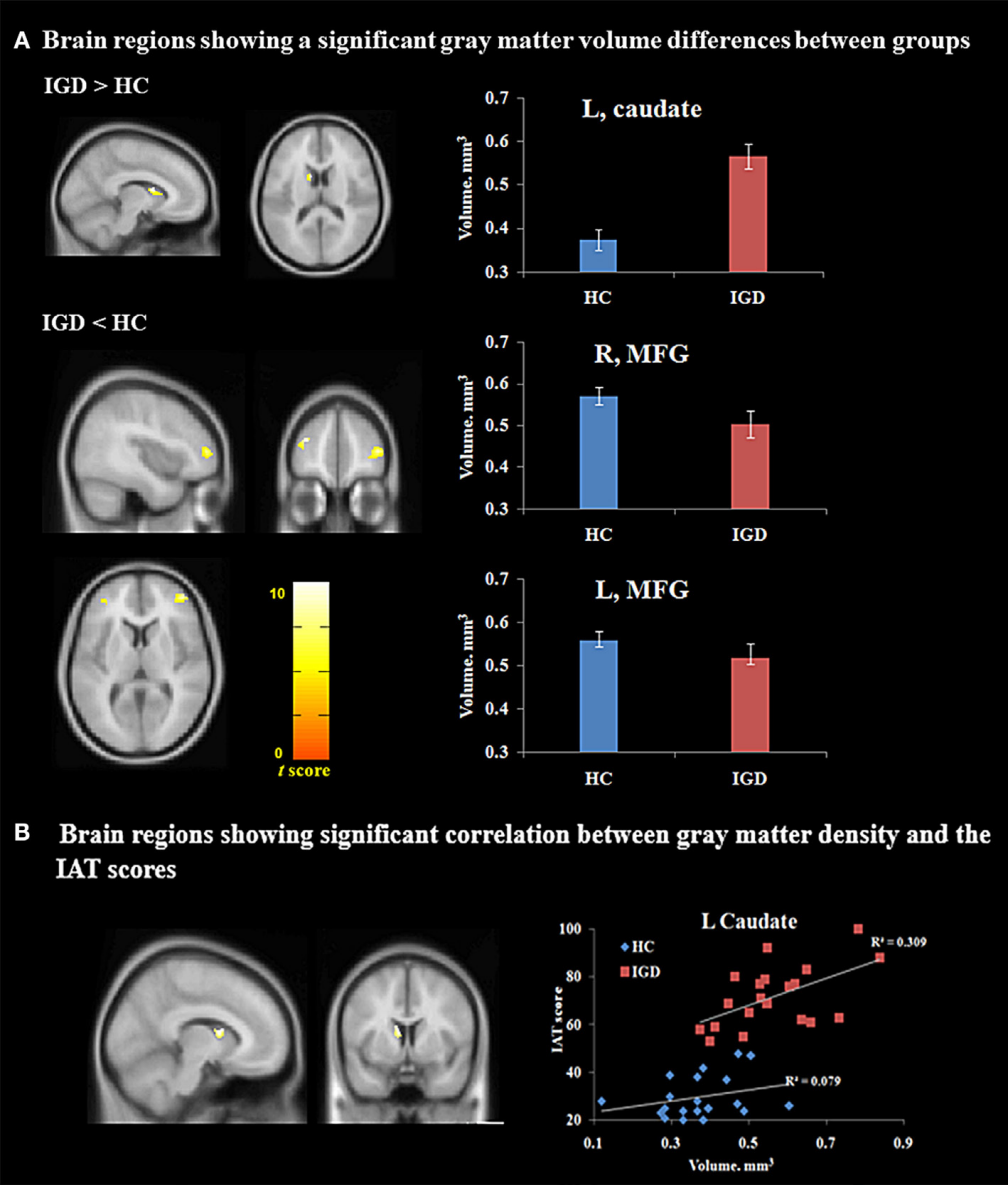
Voxel-based morphometry (VBM) analysis. **(A)** Different gray matter volumes between the IGD group and HC (*p* < 0.05, false discovery rate-corrected) (MNI coordinates: L caudate, −8, 14, 10; R MFG, 44, 51, 8; L MFG, −37, 45, 20). **(B)** VBM correlation analysis (*p* < 0.01) (MNI coordinates: L caudate, −9, 8, 15). Abbreviations: HC, healthy controls; IAT, Internet addiction test; IGD, Internet gaming disorder; L, Left; MFG, middle frontal gyrus; R, right; MNI, Montreal Neurological Institute.

As depicted in Table 2 and Figure 1A, the results of the VBM analysis show that individuals with IGD had reduced GM volume in the bilateral middle frontal cortex [Brodmann area (BA) 10] (right: *t* = 4.82, left: *t* = 4.30, *p* < 0.05, FDR corrected) and significantly increased GM volume in the left caudate nucleus (*t* = 5.37, *p* < 0.05, FDR corrected), compared with HC. After controlling for the effect of gaming activity, the GM volumes of the bilateral middle frontal cortex [right: *F*(1, 38) = 5.58, *p* < 0.05, $\eta_{\text{p}}^{2}=0.22$, left: *F*(1, 38) = 5.31, *p* < 0.05, $\eta_{\text{p}}^{2}=0.21$] and the left caudate nucleus [*F*(1, 38) = 6.59, *p* < 0.05, $\eta_{\text{p}}^{2}=0.25$] were significantly different between two groups.

**Table 2:** Regional gray matter (GM) differences between the IGD group and HC reveal a positive correlation with IGD severity.

Brain region	MNI coordinates			*t*_max_	Cluster size (voxels)
	*x*	*y*	*z*		
**IGD > HC**					
L caudate	−8	14	10	5.37	234

**IGD < HC**					
R/L MFG (BA 10)	44	51	8	4.82	417
	−37	45	20	4.30	247

**Correlation between GM density and IAT score**					
L caudate	−9	8	15	4.91	75

*BA, Brodmann area; L, left; MNI, Montreal Neurological Institute; MFG, middle frontal gyrus; R, right; IGD, Internet gaming disorder; IAT, Internet addiction test; HC, healthy controls*.

*MNI coordinates of maximum t-scores are shown for each cluster*.

*Significance at regions of interest level, p < 0.05, false discovery rate cluster-corrected*.

For the IGD group, a significantly positive correlation was found between the GM volume in the left caudate nucleus and IGD severity (i.e., IAT scores) with excluding the extraneous variables (partial correlation *r* = 0.58, *p* < 0.01, FDR corrected) (Figure 1B), and with excluding the effect of gaming activity and other extraneous variables, these positive correlations were also found between the left caudate nucleus and the IAT scores (partial correlation *r* = 0.56, *p* < 0.05). A significantly negative correlation was observed between the middle frontal volume and impulsiveness as measured using Barrett’s Impulsiveness Scale (partial correlation *r* = 0.39, *p* < 0.05, FDR corrected) and this correlation was not shown after excluding the effect of gaming activity (*p* > 0.05). However, no brain area showed a significant association with the BDI scores (*p* > 0.05, FDR corrected).

In HC, no significant relationship was found between any psychological variables (i.e., IAT, BIS, and BDI scores) and the GM volume for any brain area (*p* > 0.05, FDR corrected).

### Functional Connectivity Analysis

In individuals with IGD, the left caudate was functionally connected with various brain regions, including the bilateral thalamus, putamen, posterior cingulate cortex, precuneus, pallidum, accumbens, anterior cingulate cortex, superior occipital cortex, frontal pole, superior frontal cortex, middle frontal cortex, and orbitofrontal cortex (height threshold, *p* < 0.001, uncorrected; cluster threshold, *p* < 0.05, FDR corrected). Among HC, the left caudate nucleus was functionally connected to the bilateral thalamus, putamen, posterior cingulate cortex, pallidum, accumbens, anterior cingulate cortex, orbitofrontal cortex, superior frontal cortex, middle frontal cortex, and medial frontal cortices (height threshold, *p* < 0.001, uncorrected; cluster threshold, *p* < 0.05, FDR corrected).

**Figure 2:**
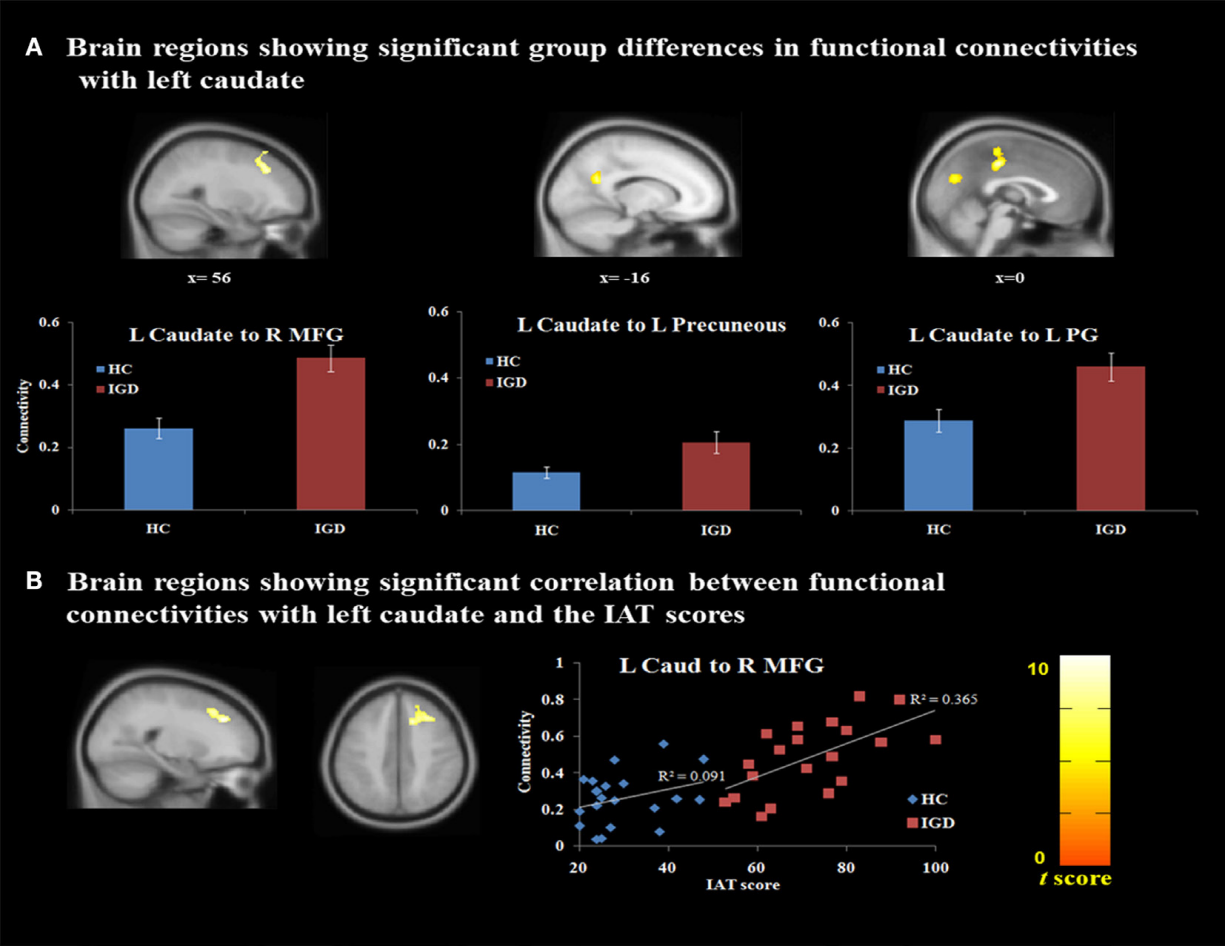
Functional connectivity analysis. **(A)** Different brain connectivity between the IGD group and HC (*p* < 0.05, FDR corrected) (MNI coordinates: L caudate, −9, 8, 15; R/L PCG, 0, -28, 44; R MFG, 35, 12, 40; L precuneus, −16, −56, 26). **(B)** Correlation analysis between IGD severity and the functional connectivity value (*p* < 0.05, FDR corrected) (MNI coordinates: L caudate, −9, 8, 15; R MFG, 22, 36, 34). Abbreviations: HC, healthy controls; IAT, Internet addiction test; IGD, Internet gaming disorder; L, Left; MFG, middle frontal gyrus; PG, postcingulate gyrus; R, right; FDR, false discovery rate; MNI, Montreal Neurological Institute; PCG, posterior cingulate gyrus.

As shown in Table 3 and Figure 2A, increased functional connectivity was observed between the left caudate and bilateral posterior cingulate gyrus (PCG) (BA 31) (*t* = 5.97, *p* < 0.05, FDR corrected), right middle frontal gyrus (MFG) (BA 8) (*t* = 11.39, *p* < 0.05, FDR corrected), and left precuneus (BA 31) (*t* = 5.48, *p* < 0.05, FDR corrected) within individuals with IGD relative to controls. After controlling for the effect of gaming activity, these increased connectivities among IGD subjects were shown in the left caudate and bilateral PCG [*F*(1, 38) = 6.27, *p* < 0.05, $\eta_{\text{p}}^{2}=0.23$], right MFG [*F*(1, 38) = 13.08, *p* < 0.001, $\eta_{\text{p}}^{2}=0.39$], and left precuneus [*F*(1, 38) = 7.22, *p* < 0.05, $\eta_{\text{p}}^{2}=0.26$].

**Table 3:** Differences in functional connectivity a between the IGD group and HC reveal a positive correlation with IGD severity.

Seed ROI	Connected region	MNI coordinates			*t*_max_	Cluster size (voxels)
		*x*	*y*	*z*		
**IGD > HC**						
L caudate	R/L PCG (BA 31)	0	-28	44	5.97	391
	R MFG (BA 8)	35	12	40	11.39	506
	L precuneus (BA 31)	−16	−56	26	5.48	381

**Correlation between functional connectivity and IAT score**						
L caudate	R MFG (BA 8)	22	36	34	6.26	446

*BA, Brodmann area; HC, healthy controls; IGD, Internet gaming disorder; L, left; MFG, middle frontal gyrus; MNI, Montreal Neurological Institute; PCG, posterior cingulate gyrus; R, right; ROI, region of interest*.

*Cluster level FDR corrected, p < 0.05, the initial height threshold is p < 0.001*.

Within the IGD group, a significantly positive correlation was found between IGD severity (i.e., IAT scores) and the functional connectivity of the left caudate nucleus with the right middle frontal cortex with excluding the extraneous variables (partial correlation *r* = 0.61, *p* < 0.01, FDR corrected) (Figure 2B). After excluding the effect of gaming activity, a significant positive correlation was also found between the severity of IGD and functional connectivity of left caudate nucleus with the right middle frontal cortex with excluding the effect of gaming activity and other extraneous variables (partial correlation *r* = 0.63, *p* < 0.01).

No significant relationship between the other psychological variables (i.e., BIS and BDI scores) and the connectivity of the left caudate nucleus with the right middle frontal cortex was noted in the IGD group (*p* > 0.05, FDR corrected). Among the HC, there was no significant correlation between the psychological variables (i.e., IAT, BIS, and BDI scores) and the connectivity of the left caudate nucleus with other brain areas.

### Correlation Analysis Between Brain Structure and Functional Connectivity

There was no significant correlation between GM volume and functional connectivity within the caudate nucleus (*r* = 0.08, *p* > 0.05).

## Discussion

This study investigated the structural and functional neural correlates of IGD by combining structural MRI and resting-state fMRI analyses. Consistent with previous studies on the comorbid psychopathology of excessive Internet use (39, 40), we observed that individuals with IGD had higher levels of depression and impulsiveness. The neuroimaging results show that the IAT score is positively linked to both the GM volume in the left caudate nucleus and the value of functional connectivity between the left caudate nucleus and the right middle frontal cortex. Interestingly, the GM deficits in the left caudate nucleus and the altered resting-state connectivity between the left caudate nucleus and the right middle frontal cortex were shown after controlling for the effect of gaming activity among individuals with IGD. However, we did not observe a link between the structural and functional alterations. These findings suggest that the left caudate nucleus is an important region in the pathogenesis of excessive Internet gaming behavior.

We found structural alterations in the left caudate nucleus of individuals with IGD relative to controls, and GM volume in the left caudate nucleus was positively related to IGD severity. These results are consistent with previous structural studies of addiction, including studies on substance addiction (41, 42), gambling addiction (43), and IGD (25, 44). The caudate nucleus is an essential part of the striatum and plays a pivotal role in reward-based behavioral learning. Moreover, the caudate nucleus is intricately linked to pleasure and motivation, and to the development and maintenance of addictive behaviors (45–47). Several studies have reported that IGD is associated with abnormalities in the striatum, specifically the caudate nucleus. For example, Kim et al. (3) and Hou et al. (48) reported reduced levels of dopamine D2 receptor and dopamine transporter in the caudate among individuals with IGD, which suggests that IGD is associated with lower levels of dopaminergic activity in the brain reward pathways, similar to other addictive disorders. Moreover, a previous fMRI study by our group using a decision-making task has revealed that higher activation in the left caudate was associated with choosing risky options, which provides more insight into the involvement of the left caudate nucleus in neural functions of reward prediction and anticipation (11). Together, these findings, suggest that reduced GM volume in the left caudate nucleus may contribute to increased sensitivity of reward anticipation in individuals with IGD; the left caudate nucleus may thus be part of the relevant functional circuitry associated with IGD.

To investigate the relationship between structural alterations and aberrant functional connectivity, we performed a seed-based resting-state functional connectivity analysis. The functional connectivity analysis with a seed in the left caudate nucleus revealed that the right middle frontal cortex (i.e., the DLPFC) was positively correlated with IGD severity, indicating that individuals who were more preoccupied with Internet gaming had stronger connectivity between the left caudate nucleus and the right DLPFC. The area shown in the VBM result did not exactly correspond to the area shown in the rs-fMRI result. The area shown in the VBM and rs-fMRI results was BA 10 and 8, respectively, and the overlapping area is merely partial. However, all of the area is included in DLPFC. The DLPFC-striatal circuit is a key part of the dopamine reward circuit and is strongly implicated in executive functions such as planning, organization, set shifting, and attention (49). Dysfunction of this network may impact the maintenance of addiction by reducing the ability to regulate the integration and selection of cognitive and goal-motivated behavior (49). Aberrant frontostriatal circuits have previously been revealed in individuals with IGD. A study on resting-state functional connectivity suggests that adolescents with Internet addiction have alterations in their frontostriatal circuits that impair affect, motivation processing, and cognitive control (50). Consistent with our results, another study showed that functional connectivity in the frontostriatal network was positively associated with higher severity of Internet addiction (51). However, in contrast to the present results, other functional connectivity studies have shown that individuals with IGD have decreased functional connectivity in the frontostriatal circuit (24, 25). A recent review on neuroimaging findings in IGD also indicated inconsistent results among the studies and suggested that the altered brain is not robust and merits further investigation (28). The discrepancy between these findings may be due to demographic or clinical factors such as sex, age, durations of illness, or treatment-seeking status. Numerous neuroimaging studies have also indicated that the caudate nucleus and DLPFC are closely involved in video game playing (52–54). These studies have demonstrated that the left striatum and DLPFC plasticity is related to the amount of game playing/training in non-addicted subjects. In the study, to identify that the alteration in these regions are more related to IGD characteristic including addictive characteristic or more linked to gaming activity, we conducted further analysis after controlling for the effect of gaming activity (i.e., the average gaming hours). The results of the further analysis clearly showed the differences between the groups. Therefore, the alteration in these areas may be more related to the IGD characteristics rather than gaming activity. Taken together, regardless of such inconsistencies, the findings to date suggest that the dysfunction of the frontostriatal circuit during the resting state and its relationship with IGD severity may be associated with inappropriate behavioral choices, such as seeking Internet use despite the negative consequences.

Several limitations of this study should be noted. First, due to the cross-sectional nature of the study, cause-and-effect relationships are unclear. Future studies should identify longitudinal effects on IGD. Second, we limited our study cohort to males of 20–27 years of age, and caution should thus be exercised when generalizing the results of our study to the general population, also considering the small sample size. Third, future studies may consider measuring the time since IGD diagnosis to explain any significant variability in neural functioning. Finally, there is some contradiction between our findings and the other showing increased and decreased functional connectivity in the frontostriatal circuit. Therefore, the results should be interpreted with caution and further studies under the same conditions (i.e., demographic characteristics or with clinically similar participants) are needed to explain the contradiction (24, 25, 28).

In conclusion, this study reveals structural alterations of the caudate nucleus and dysfunctionalities of the frontostriatal networks in individuals with IGD. More importantly, both types of alterations were associated with IGD severity. Our results suggest that the left caudate nucleus plays a key role in the pathogenesis of IGD and that IGD and substance abuse share similar neural mechanisms.

## Ethics Statement

All participants provided their written informed consent after being thoroughly informed about the details of the experiment. The Chungnam National University Institutional Review Board (IRB) approved the experimental and consent procedures (approval number: P01-201602-11-002). All participants received financial compensation (50 US dollars) for their participation.

## Author Contributions

JWS contributed to conception and experimental design, or acquisition of data, or analysis and interpretation of data, and JHS contributed substantially to interpretation of data and drafted the article or revised it critically for important intellectual content.

## Conflict of Interest Statement

The authors declare that the research was conducted in the absence of any commercial or financial relationships that could be construed as a potential conflict of interest.

## Abbreviations

BIS, Barratt Impulsiveness Scale-II; BDI, Beck Depression Inventory; DLPFC, dorsolateral prefrontal cortex; FDR, false discovery rate; fMRI, functional magnetic resonance imaging; GM, gray matter; IAT, Internet addiction test; IGD, Internet gaming disorder; VBM, voxel-based morphometry; MNI, Montreal Neurological Institute; WM, white matter.
